# Typhus Fever

**Published:** 1918-09

**Authors:** A. L. Lincecum

**Affiliations:** El Campo, Texas


### BUFFALO MEDICAL JOURNAL

Yearly Volume 74 SEPTEMBER, 1918 Number 2

### ORIGINAL ARTICLES

The right is reserved to decline papers not dealing with practical medical and surgical subjects, and such as might offend or fail to interest readers. Contributors are solely responsible for opinions, methods of expression and revision of proof.

### Typhus Fever.

DR. A. L. LINCECUM, El Campo, Texas; Captain M. R. C.

Typhus, ship, prison or spotted fever, exists in practically all the civilized countries of the world open to our commerce and from which soldiers are likely to be drawn to the western front. It is an acute infectious fever, unquestionably due to a specific germ but the exact organism has not been demonstrated and it would be idle to speculate as to its nature, whether bacterial or protozoic. However, as with yellow fever, while we do not know the exact organism we do know the method of transmission, namely by the bite of the body louse, apparently not by other species. The bed bug has also been incriminated with some degree of probability and it is possible that other insects may serve as carriers but the disease is no more contagious than yellow fever. In fact, after thorough delousing, I have had patients cared for in houses occupied by persons free from the disease, without their developing it.

Typhus is endemic in Mexico where frequent epidemics occur in winter. It is from this source that the disease is mainly introduced into the southern and southwestern states. At times it is traced or probably due to individual Mexicans or returning Americans who have crossed the border surreptitiously without inspection and delousing. For example, a Mexican was found by a police officer reeling along the streets of a Texas town, was arrested and put into the jail. He died within a few days and was buried without having any special attention directed to his case but, whether he was actually suffering from typhus and whether his arrest for drunkenness was warranted or due to the weakness of disease, or whether he merely served to convey infected lice to the jail, seven other prisoners shortly after his death, developed the disease.

The incubation period of the disease is about 12 days but the danger may persist almost indefinitely in premises in which lice have not been destroyed. Two special sources of menace to the U. S. may be mentioned. Mail bags loaded at, say, Mexico City are shipped under guard of Indians who are practically universally lousy and who sleep on the bags. During the disturbances which have existed in Mexico for several years, various leaders have held back freight cars from the U. S. to transport troops, and lice have been found in these cars even after they had been sealed up for three or four weeks. Paradoxically, the persistence of lice under such circumstances, is due to their habit of destroying one another. They are markedly cannibalistic and it is literally a case of the survival of the fittest. For example, in one experiment, three species of lice and several other insects were placed in a bottle. In the course of a few days, only the most vigorous of one species of lice remained. It is obvious, from the almost universal reports of infestment with lice in the trenches, that it is largely a matter of accident that typhus has not been prominent on the western front as it was in the Balkans and an appreciation of the means of conveyance and of prevention, as well as the ability to recognize the disease early, are matters of military preparedness.

A typic, severe case of typhus begins almost without prodromes; with sudden chill, agonizing pains in the back, head and extremities so that it might well be mistaken for meningitis. Indeed, the differential diagnosis must depend upon the results of examination of spinal fluid withdrawn by lumbar puncture. Muttering delirium or maniacal symptoms may also occur. Or, there may be bronchitis with cough. Again, the disease may be marked by obstinate vomiting and alimentary disturbances. Haemorrhages may occur, either assuming the form of haemoptysis or haematemesis. As in various other general infections, haemorrhagic cases are likely to be malignant. Superimposed typic temperature charts of typhoid and typhus resemble each other greatly at first, though the rise in typhus is usually more rapid and a temperature of 105° is quite uniformly reached on the second or third day, while the morning remissions are less than in typhoid, being very close to one degree below the evening temperature. However, the tongue also resembles that of typhoid in the general initial redness and subsequent light coat and the early differential diagnosis may be extremely difficult; indeed, if typhus is not known to exist, an error in the early stages of the first case encountered is almost inevitable. The Widal reaction cannot be depended upon early as it is rarely present in typhoid till the close of the first week while, in persons already inoculated, only cultures can be relied on to establish the differential diagnosis against typhoid. Later, however, the typhus tongue becomes brown and, about the fourth of fifth day, it has the appearance of being varnished. There is tremor of the protruded tongue and, indeed, of the muscles generally. Thus, according to circumstances, further confusion with either typhoid or meningitis may occur.

The most characteristic point in typhus is the eruption which appears from the second to the fifth day. It is usually first noticed on the abdomen but, on turning the patient over, it will also be seen on all posterior surfaces subjected to most pressure. Thus the patient should always be carefully inspected after turning over. The eruption is, at first, a bluish, mottled discoloration, under the skin, later becoming macular and, according to many authorities, papular, but I have never observed this appearance either in this country or in Mexico where I visited several hospitals in order to study the disease, nor did the Mexican physicians describe the papular stage. Without denying that papules may form, I am inclined to believe that their description has been by anticipation, as the development of a papule from a macule is so probable a priori. About the fourth or fifth day of the eruption, it becomes distinctly petechial, being lighter colored—or, perhaps it would be better to say dimmer—on parts exposed to the air, than on those kept covered by clothing. It will be noted that the eruption includes the haemorrhagic factor already noted as marking special clinical types. Obviously, in certain cases, there may be a confusion with haemorrhagic small pox, especially if typhus attacks an unvaccinated person when small pox is prevalent.

There is almost invariably constipation and a tendency to retention of urine in typhus, incontinence being observed, however, in cases that are rapidly fatal.

After a few days, the temperature chart becomes much more significant diagnostically, the temperature oscillating between 104° and 105° quite regularly for just about 16 days when there is a critical fall, and this is the time of greatest danger in cases that have not overwhelmed the patient within the first few days. Heat and stimulants should be freely used at this stage. It may be questioned whether the more gradual fall of temperature in typhoid is not an essentially conservative factor.

If patients live long enough, remembering that promptly fatal cases do not have time to develop complications and that they are overwhelmed by the general intoxication of the infection without even the development of a morbid anatomy sufficient to explain death, the most frequent complication is broncho-pneumonia. Gangrene of the lungs may occur. There may be suppurating otitis media or various other septic processes and gangrene of the fingers and toes is especially characteristic, according to some, though I must confess that I never saw gangrene in the Mexican hospitals and the general opinion there was that the disease could be pretty sharply divided into maligant, rapidly fatal, cases, and those that go on to crisis at about the 16th day, when recovery or death depends largely on the resistance of the patient and the efficiency of supporting treatment, bearing in mind that a large proportion of the poorer classes of Mexico are insufficiently fed and, even if not literally lacking food, deficient in proteid and accustomed to a badly balanced diet. Many Mexican epidemics have a mortality of 60% and one at San Luis Potosi in 1916 had a mortality of about 87%, explained by an almost total local deficiency in corn and beans and general recourse to whatever foods low in dietetic value could be obtained. Rare complications are nephritis and meningitis.

It may further be remarked that typhus may resemble dengue which is a disease of marked symptoms but almost devoid of danger to life. I recall an isolated case in Texas in which physicians insisted on this diagnosis while a few of us were equally certain that the case was one of typhus. On the fatal termination, some of our opponents rather reluctantly admitted that we might possibly have been right.

The first attempts that we made to prevent the introduction of infection across the border, by lice, were rendered difficult by lack of available funds and were carried out in a somewhat crude, though quite effective way. Large dry-goods boxes were made practically air-tight by pasting paper over the cracks and covering them with tar paper and fumigation with hydrocyanic acid gas was employed, a small charge being made for baggage and for freight or box cars. At first, the space available was sufficient only to fumigate six or eight suit cases at a time. As funds accumulated, a more elaborate steam apparatus was installed.

It is obvious that this method involves danger and can not be employed for delousing human beings. In attacking this problem, it is important to remember that the lice and especially the eggs are to be found especially on hair, the female louse secreting a sort of cement which glues the eggs to the shaft of the hair. This cement is dissolved by acetic acid while the adult insect, like most others, is destroyed by kerosene. Hence we adopted the simple method of spraying with equal parts of vinegar and kerosene, using a pressure atomizer, though thorough rubbing may be practiced if an atomizer is not available. Especially in the case of women, the head was wrapped in a towel for 15 minutes and then a hot shower was used. Xylol and soap, turpentine, oil of cinnamon, even iodoform, and similar substances are reported to have been used in the eradication of lice by the French but none of these means are so effective.

Steam naturally suggests itself for the destruction of lice and their ova in clothing and some such device as the Serbian barrel may be employed but it is difficult to secure a sufficiently high temperature to kill the lice without damaging many articles of clothing. Here again, it is of practical importance to remember that just as lice on the body favor the hairy surfaces, the sites of election in clothing are the seams or similar niduses, as under labels. They may be exterminated, therefore, by sponging and running hot irons along these places.

To prevent infestation with lice under the conditions of trench warfare is a very difficult task, as it depends on universal cleanliness. It has been said that lice will not lay eggs in silk underwear and this I have found to be true but it may also be said with confidence that lice will infest persons without regard to what underwear they select or even if they wear none at all. Some years ago, a commission went from this country to study typhus in Mexico. One of the members died of the disease in Mexico. Another, who had sought to protect himself by wearing silk underwear and by changing it and bathing three times a day and by wearing rubber gloves when investigating cases, came home sick with typhus but eventually recovered. As, even in delousing stations, the lice are apt to fall to the floor and crawl onto another person, some precaution must be observed. We found that the simplest and best was to apply kerosene to the floor. While this does not immediately kill the lice it seems to paralyse them so that they cannot crawl to another person and they soon die.

The treatment of typhus is entirely symptomatic. In Mexico, I found a French proprietary proteid silver combination, supplied in ampoules, largely used hypodermatically but, so far as I could observe, the patients thus treated died as frequently as the average.

One important point in the clinical side of typhus is the entire lack of correspondence between pulse and temperature and, one might almost say, the lack of prognostic value of either. The pulse may remain very near 80, usually very rapid however, throughout the entire febrile period, with the temperature varying only from about 104° to 105°. Even at the time of the dangerous critical fall of temperature, the pulse does not necessarily show much change though, before death occurs, it becomes rapid and feeble. I can easily count a pulse of 140 but I have had cases of typhus in which the pulse could not be counted, though this was not entirely due to the rapidity but to the extremely low blood pressure so that the pulse was merely a wave instead of a distinct pulsation.

In emphasizing the close analogy to yellow fever, I neglected to state that in the case of typhus, there is an important difference: There is nothing to indicate that any period of incubation in the insect is necessary before it can transmit the disease. One important point in the control of the disease is that mere quarantine or isolation is of little value. Even if a house is emptied of all other inmates, the infected lice will persist in the premises almost indefinitely. Thus, thorough delousing of the patient, his associates and the premises is absolutely necessary. This may necessitate the removal of the patient to allow the delousing of the premises but, if lice are eradicated, no danger of transmission exists, whether the patient is treated on the same premises or elsewhere and isolation is not necessary.

One peculiarity of the Mexican body lice is that they are nearly black but this is probably due merely to the well established tendency of the insect to assume a color corresponding approximately to the complexion of the host. For example, in Mexico, I noticed a blond American whose lice were of the usual light color observed in the United States.

One physician, as a precaution against lice and other vermin, with no special reference to typhus danger, had a thick muslin suit made, with reinforcement of the openings after the manner of the tongues of shoes, using adhesive plaster to close the access at the wrists and ankles.

---

**Intra-Ocular Foreign Bodies.** Lapersonne, Le Prog. Med., Mch. 2, emphasizes the danger of unilateral or total blindness from even minute foreign bodies, the prophylactic value of masks and spectacles and the importance of early extraction after careful examination.

---

**Simple Blood Transfusion.** P. Ameuille, Le Prog. Med., Mch. 2, favors the method of Jeanbrau and Rosenthal. Blood is taken from a donor, from a vein, received into a flask containing sodium citrate and kept in readiness in the incubator, if necessary for several days. It is then injected into a vein.

